# Correlation Between Postoperative Pain Intensity and Perioperative Blood Loss After Total Knee Arthroplasty

**DOI:** 10.1155/aort/6028094

**Published:** 2025-12-15

**Authors:** Panapol Varakornpipat, Sirikarn Tananoo, Akaworn Mahatthanatrakul, Settawut Phanyosri, Sasawat Ratanapises, Artit Laoruengthana

**Affiliations:** ^1^ Department of Orthopaedics, Faculty of Medicine, Naresuan University, Phitsanulok, Thailand, nu.ac.th

**Keywords:** blood loss, postoperative pain, total knee arthroplasty

## Abstract

**Introduction:**

Postoperative pain following total knee arthroplasty (TKA) can exacerbate sympathetic activity, elevate blood pressure, and potentially increase blood loss. However, the relationship between these factors remains unclear. This study evaluates the correlation between postoperative pain intensity and perioperative blood loss.

**Methods:**

A retrospective analysis was conducted on 405 unilateral TKAs, stratified by average visual analog scale (VAS) in the first 24 h postoperatively into mild (VAS 0–3), moderate (VAS > 3–6), and severe (VAS > 6–10) pain intensity groups. The primary outcomes were the effect of pain intensity on mean calculated blood loss (CBL) and risk of blood transfusion. Secondary outcomes included length of hospital stay (LHS) and complications. Multivariable regression analysis was employed.

**Results:**

Adjusted mean differences in CBL and LHS were not significantly different among the pain intensity groups. However, severe pain intensity was associated with a higher risk of blood transfusion (risk ratio: 1.92, *p* = 0.068). Preoperative hemoglobin (Hb) was the only protective factor against blood transfusion; each 1 g/dL increase in preoperative Hb reduced the blood transfusion risk by 61% (risk ratio: 0.39, *p*  <  0.001). Additionally, the severe pain intensity group had the highest incidence of overall complications (13.3%) observed during 180 days post‐TKA (*p*  <  0.01).

**Conclusion:**

Patients experiencing severe pain intensity in the first 24 h after TKA tend to have a higher risk for blood transfusion and a significantly higher risk of overall complications. Thus, optimizing pain control is important to enhance recovery for current clinical pathways of TKA.

## 1. Introduction

Total knee arthroplasty (TKA) is frequently associated with significant blood loss, and excessive perioperative blood loss may necessitate allogenic blood transfusion [1]. Patients who require blood transfusions are at increased risks of allergic reactions, hemolytic transfusion reactions, prolonged hospital stays, superficial and deep wound infections, disease transmission, and, rarely, acute lung injury [2]. Several modifiable and nonmodifiable factors have been identified to influence or associate with blood loss after TKA. These factors include advanced age, female gender, high body mass index (BMI), preoperative anemia, extensive soft tissue dissection, multiple bone cuts, and prolonged operative time [3–5].

Theoretically, pain pathway stimulation can result in tachycardia and increased blood pressure, exacerbating sympathetic activity and indirectly increasing blood loss [6, 7]. Excessive postoperative pain might also influence perioperative blood loss through various mechanisms, such as increased muscle contraction during motion, resulting in elevated venous blood pressure [7, 8]. Additionally, postoperative pain is significantly associated with delayed recovery after TKA and increased stress on the cardiovascular system and may contribute to complications such as ischemic cardiac events and myocardial insufficiency [8–10]. Thus, effective management of pain and bleeding during the perioperative period after TKA is a fundamental concern.

However, few studies explored the link between postoperative pain intensity and perioperative blood loss, and the existing evidence remains inconsistent [7, 11, 12]. Hence, the objective of this study was to assess the relationship between postoperative pain intensity and perioperative blood loss, as well as to evaluate the association between pain intensity and complications, including length of hospital stay (LHS), in patients who underwent TKA. We hypothesized that patients who experience severe pain intensity after TKA might have higher perioperative blood loss and longer LHS compared to those with mild pain levels.

## 2. Materials and Methods

This retrospective observational cohort study was conducted at a single institution. Patients aged over 45 years with knee osteoarthritis who underwent primary unilateral TKA consecutively performed by a single surgeon between 2020 and 2024 were included. Exclusion criteria were a history of previous knee surgery, knee infection, diagnosis of secondary osteoarthritis, congenital or acquired coagulopathy, and preoperative hemoglobin (Hb) < 9.0 g/dL, history of substance use or addiction, and stopped taking anticoagulant < 7 days before the index surgery. Patients were classified based on their average visual analog scale (VAS) for pain intensity in the first 24 h post‐TKA into three groups according to established pain level categories: mild (VAS 0–3), moderate (VAS > 3–6), and severe (VAS > 6–10) [13]. The sample size was calculated based on the mean calculated blood loss (CBL) of the mild and moderate pain intensity groups. It was estimated that a sample size of 150 patients in each group would provide 80% power to detect a difference between a mean CBL of 530 ± 10.02 in the mild pain intensity group and 533.56 ± 9.97 in the moderate pain intensity group, with a two‐sided type I error rate of 5%. The study protocol was approved by Institutional Review Board, Naresuan University (COA No. 127/2025), and was registered on Thai clinical trial registry (TCTR 20250509003) (https://www.thaiclinicaltrials.org/show/TCTR20250509003). After IRB approval, informed consent was not obtained for this study due to its retrospective nature and the lack of further contact with participants.

### 2.1. Surgical Procedure and Outcome Measurement

All patients received standardized preemptive medication, including a tranquilizer and gabapentinoid, the night before surgery. Normotensive spinal anesthesia was performed at the L2‐3 or L3‐4 intervertebral space using 14–16 mg (2.8–3.2 mL) of bupivacaine (0.5% Heavy Marcaine Spinal, Aspen, France). Adjunct multimodal pain management approaches were categorized into four groups: (A) peripheral nerve block (PNB): either femoral nerve block (FNB) or adductor canal block (ACB), (B) intraoperative local infiltration analgesia (LIA) only, (C) PNB + LIA, and (D) spinal anesthesia with intrathecal bupivacaine and 0.1–0.2 mg of morphine, PNB, and LIA. All the ACB and FNB were performed under ultrasonography guidance using 20 mL of 0.25% bupivacaine before spinal anesthesia. A prophylactic antibiotic was administered in all TKA procedures and a tourniquet inflated to 250 mmHg was applied before skin incision until skin closure.

A standard medial parapatellar arthrotomy with approximately 10 cm skin incision was performed. Proximal tibial and distal femoral bone cuts were prepared using conventional instruments with extramedullary and intramedullary reference guides, respectively. Patellar resurfacing was carried out in all patients. A bone plug occluded the femoral medullary canal, and soft tissue was balanced for appropriate flexion and extension gaps. For groups B, C, and D, LIA was administered before prosthetic implantation using 10 mL of 0.5% bupivacaine diluted to 75 mL with sterile normal saline. The anesthetic solution was applied to the medial retinaculum, quadriceps muscle, pes anserinus, retropatellar fat pad, posterior capsule, and medial/lateral meniscal remnants. Fixed‐bearing TKA prostheses were implanted with bone cement. Before arthrotomy closure, a negative pressure drain was applied and topical tranexamic acid (TXA) (15 mg/kg) was poured into the knee joint to reduce blood loss and mitigate postoperative pain [14]. The compressive dressing was applied and the drain was clamped for 3 h and discarded after 24 h postsurgery.

Postoperative protocols included intravenous patient‐controlled analgesia (PCA) with 0.5 mg morphine on‐demand boluses (5‐minute lockout) for 48 h. Oral acetaminophen (500 mg thrice daily) and a nighttime tranquilizer were administered. Intravenous ketorolac (30 mg, ketorolac tromethamine 1 mL, SiuGuan, Taiwan) was given every 8 h, and then switched by oral naproxen (250 mg twice daily), except for patients with contraindications to nonsteroidal anti‐inflammatory drugs (NSAIDs). Intravenous morphine (2 mg) was available as rescue analgesia until discharge. Postoperative rehabilitation included the use of a continuous passive motion (CPM) and early ambulation with gait aids. The dataset, recorded by junior doctors in the orthopedic department, included age, gender, BMI, American Society of Anesthesiologists (ASA) physical status classification, comorbidities (hypertension, diabetes mellitus, and dyslipidemia), preoperative and postoperative Hb levels, NSAID use, analgesic modalities, operative time, blood transfusion, and LHS.

The primary outcomes were the effect of pain intensity in the first 24 h post‐TKA on mean CBL and risk of blood transfusion. Pain intensity was measured using a 10‐cm VAS at 6, 12, 18, and 24 h postoperatively. The average of VAS in the first 24 h was stratified into mild, moderate, and severe pain intensity groups. For CBL, the patient’s total blood volume (TBV) was estimated using the equation by Nadler et al. [15]. The difference between preoperative and postoperative Hb at 48 h was applied with the Hb balance method, to determine CBL [16]. This measure was accounted for the sum of external blood loss and hidden blood loss (HBL) (Table 1) [17]. A serum Hb level below 9.0 g/dL is indicated as our blood transfusion threshold. Secondary outcomes included the effect of pain intensity on LHS and complications, as well as identifying independent factors affecting these outcomes. All the outcomes were routinely recorded by the assessors who were blinded to the analgesic modalities.

**Table 1:** Calculated blood loss and total body volume formulation [16, 17].

Parameter	Formulation
Male: TBV (mL)	(0.0003669 × height^3^ (cm)) + (32.19 × body weight (kg)) + 604
Female: TBV (mL)	(0.0003561 × height^3^ (cm)) + (33.08 × body weight (kg)) + 183
Calculated blood loss (mL)	TBV (ml) × (Hbi – Hbe)/Hbi + sum of blood products transfused (ml)

Abbreviations: TBV, total body volume; Hbi_i_ [g/dL], preoperative hemoglobin; Hbe_e_ [g/dL], postoperative hemoglobin.

### 2.2. Statistical Analysis

Statistical analysis was performed using STATA Version 17 (StataCorp LLC, Texas, USA). Fischer’s exact test, *t*‐test, and ANOVA were used to compare categorical and continuous prognostic factors among pain intensity groups. Crude primary and secondary outcomes were analyzed using univariable Gaussian regression. Multivariable regression was employed to adjust for the potential confounding factors previously described, including age, gender, BMI, ASA score, comorbidities (hypertension, diabetes mellitus, and dyslipidemia), preoperative Hb, tourniquet time, and NSAID use at 24 h [3–5, 17–19]. Mean CBL and LHS were analyzed using Gaussian regression, while the risk of blood transfusion was analyzed using risk ratio (multivariable Poisson) regression. Statistical significance was set at *p*  <  0.05.

## 3. Results

The study cohort comprised 405 patients: 174 in the mild pain intensity group, 186 in the moderate pain intensity group, and 45 in the severe pain intensity group. No significant differences were observed among the three groups in age, BMI, ASA score, or preoperative Hb. The mild pain intensity group had a higher percentage of male patients compared to the moderate and severe pain intensity groups. The moderate pain intensity group had the longest operative time. Patients in the severe pain intensity group exhibited a higher prevalence of dyslipidemia, lower proportion of NSAID use, highest morphine consumption in the first 24 h, and lesser arc of knee flexion compared to the other groups (Table 2).

**Table 2:** Demographic and perioperative characteristics.

	Mild pain intensity (*N* = 174)	Moderate pain intensity (*N* = 186)	Severe pain intensity (*N* = 45)	*p* value
Age (years)	66.95 ± 7.61	66.19 ± 7.58	65.78 ± 7.80	0.139
Gender, *N* (%)				0.034^a^
Male	40 (22.99)	24 (12.90)	6 (13.33)	
Female	134 (77.01)	162 (87.10)	39 (86.67)	
BMI (kg/m^2^)	26.93 ± 4.41	26.88 ± 4.20	27.25 ± 4.28	0.329
ASA, *N* (%)				0.949
1–2	87 (50.00)	90 (48.39)	23 (51.11)	
3	87 (50.00)	96 (51.61)	22 (48.89)	
Comorbidities, *N* (%)				
Hypertension	124 (73.37)	131 (71.58)	37 (82.22)	0.367
Diabetes mellitus	44 (26.04)	34 (18.58)	8 (17.78)	0.209
Dyslipidemia	84 (49.70)	105 (57.38)	32 (71.11)	0.030^a^
NSAID use, *N* (%)	159 (91.38)	152 (81.72)	36 (80.00)	0.012^a^
Preoperative Hb (g/dL)	12.50 ± 1.29	12.30 ± 1.25	12.35 ± 1.22	0.333
Operative time (min)	62.37 ± 11.07	66.35 ± 12.79	63.22 ± 11.76	0.006^a^
Morphine use 24 h (mg)	8.13 ± 9.12	13.36 ± 8.60	18.38 ± 12.95	< 0.001^a^
Knee flexion at 24 h postoperative (degree)	61.09 ± 18.95	59.77 ± 19.49	56.53 ± 21.42	0.037^a^

*Note*: Values are shown as mean ± SD, except gender, ASA, comorbidities, and NSAID use which are shown as *n* (%).

Abbreviations: SD, standard deviation; *N*, numbers; BMI = body mass index; kg/m^2^ = kilogram/meter^2^; ASA, American Society of Anesthesiologists physical status classification; NSAIDs, nonsteroidal anti‐inflammatory drugs; Hb, hemoglobin; g/dL, gram/deciliter; min, minutes; mg, milligram.

^a^Statistical significance.

Stratification of pain intensity levels according to analgesic modalities revealed significant differences in the overall proportion of pain intensity among analgesic modalities (*p*  <  0.001). The PNB + LIA + ITM modality had the highest proportion of mild pain intensity (76.19%) and the lowest proportion of severe pain intensity (3.57%). Conversely, severe pain intensity was more frequently observed in PNB and LIA modalities (14.00% and 14.29%, respectively) (Table 3). Mean VAS scores across different analgesic modalities are presented in Table 4.

**Table 3:** Stratification of pain intensity level according to analgesic modalities.

	Mild pain intensity *N* (%)	Moderate pain intensity *N* (%)	Severe pain intensity *N* (%)	*p* value
PNB (*N* = 100)	24 (24.00)	62 (62.00)	14 (14.00)	< 0.001^a^
LIA (*N* = 77)	29 (37.66)	37 (48.05)	11 (14.29)	
PNB + LIA (*N* = 144)	57 (39.58)	70 (48.61)	17 (11.81)	
PNB + LIA + ITM (*N* = 84)	64 (76.19)	17 (20.24)	3 (3.57)	

Abbreviations: *N*, numbers; PNB, peripheral nerve block; LIA, local infiltration analgesia; ITM, intrathecal morphine.

^a^Statistical significance.

**Table 4:** Mean VAS according to analgesic modalities.

Analgesic modalities	Mean VAS (mean ± SD)	Compared to other analgesic modality mean difference (*p* value)		
		PNB + LIA + ITM	PNB + LIA	LIA
PNB (*N* = 100)	5.01 ± 1.99	−2.60 (< 0.001)	−0.75 (0.040)	−0.34 (1.000)
LIA (*N* = 77)	4.68 ± 2.03	−2.26 (< 0.001)	−0.41 (1.000)	
PNB + LIA (*N* = 144)	4.27 ± 2.17	−1.85 (< 0.001)		
PNB + LIA + ITM (*N* = 84)	2.41 ± 2.16			

*Note*: One‐way ANOVA was used to compare mean VAS among analgesic modalities, followed by Bonferroni post hoc tests for pairwise comparisons. *p* value < 0.5 = statistical significance.

Abbreviations: *N*, numbers; PNB, peripheral nerve block; LIA, local infiltration analgesia; ITM, intrathecal morphine; ITM, intrathecal morphine; mean VAS, mean visual analog scale in the first 24 h.

Crude analysis using univariable regression showed no significant correlation between pain intensity level and either primary or secondary outcomes (Table 5). Multivariable Gaussian regression analysis for CBL demonstrated no significant difference in adjusted mean CBL between pain intensity groups. Factors significantly correlated with CBL were operative time (each additional minute increased blood loss by 2.67 mL, *p* = 0.02) and preoperative Hb (each 1 g/dL increase was associated with 149 mL additional blood loss, *p*  <  0.001). Pain intensity level did not significantly affect the adjusted mean difference of LHS. NSAID use was the only factor that significantly reduced LHS by 0.35 days (*p* = 0.031) (Table 6). Multivariable Poisson regression analysis of blood transfusion risk revealed that patients experiencing severe pain intensity in the first 24 h tended to have an increased blood transfusion risk (risk ratio = 1.92, *p* = 0.068). Preoperative Hb was a protective factor, with each 1 g/dL increase reducing blood transfusion risk by 61% (risk ratio = 0.39, *p*  <  0.001) (Table 6).

**Table 5:** Univariable analysis for primary and secondary outcomes.

	Mild pain intensity	Moderate pain intensity	*p* value	Severe pain intensity	*p* value
Calculated blood loss (mL), mean (95% CI)	531.91 (484.31–579.50)	520.17 (474.02–566.32)	0.728	546.92 (453.60–640.24)	0.778
Blood transfusion risk, risk ratio (95% CI)	Ref.	1.32 (0.74–2.39)	0.343	1.59 (0.70–3.60)	0.264
Length of stay (days), mean (95% CI)	4.90 (4.74–5.07)	4.88 (4.72–5.03)	0.830	4.76 (4.43–5.08)	0.428

Abbreviations: mL, milliliter; 95% CI, 95% confidence interval; Ref., reference.

**Table 6:** Multivariable Gaussian regression analyses for calculated blood loss and length of hospital stay, and rate ratio regression analysis for evaluating the risk of blood transfusion.

Prognostic factors	Calculated blood loss Mean difference (95% CI)	*p* value	Length of hospital stay Mean difference (95% CI)	*p* value	Blood transfusion risk Risk ratio (95% CI)	*p* value
Pain intensity						
Mild	Ref.		Ref.		Ref.	
Moderate	11.24 (−45.14, 67.61)	0.695	−0.03 (−0.27, 0.22)	0.827	1.11 (0.63, 1.99)	0.712
Severe	31.54 (−54.43, 117.51)	0.471	−0.17 (−0.54, 0.20)	0.377	1.92 (0.95, 3.88)	0.068
Female gender	13.60 (−62.56, 89.77)	0.726	0.11 (−0.22,0.44)	0.507	1.05 (0.46, 2.37)	0.899
Age	0.45 (−3.23, 4.14)	0.808	0.01 (−0.01, 0.03)	0.188	1.00 (0.97, 1.04)	0.867
BMI	1.55 (−4.79, 7.89)	0.631	−0.01 (−0.04, 0.02)	0.498	0.94 (0.86, 1.03)	0.162
ASA class 3	14.07 (−43.13, 71.27)	0.629	0.04 (−0.21, 0.29)	0.743	1.32 (0.72, 2.43)	0.375
Hypertension	−0.07 (−63.03, 62.88)	0.998	0.00 (−0.27, 0.28)	0.975	0.64 (0.36, 1.16)	0.144
Diabetes mellitus	19.56 (−46.32, 85.43)	0.560	0.06 (−0.23, 0.34)	0.694	1.61 (0.90, 2.87)	0.108
Dyslipidemia	32.73 (−22.34, 87.81)	0.243	−0.09 (−0.33, 0.14)	0.433	1.47 (0.87, 2.49)	0.150
NSAID use	25.92 (−47.09, 98.93)	0.486	−0.35 (−0.66, −0.03)	0.031^a^	0.94 (0.51, 1.76)	0.852
Operative time	2.67 (0.44, 4.91)	0.019^a^	−0.01 (−0.02, 0.00)	0.131	1.00 (0.98, 1.02)	0.933
Preoperative Hb	148.98 (126.44, 171.51)	< 0.001^a^	−0.03 (−0.13, 0.07)	0.566	0.39 (0.33, 0.48)	< 0.001^a^

Abbreviations: BMI, body mass index; ASA, American Society of Anesthesiologists physical status classification; NSAIDs, nonsteroidal anti‐inflammatory drugs; Hb, hemoglobin; 95% CI, 95% confidence interval; Ref., reference.

^a^Statistically significant.

Complications observed during the 180‐day postoperative period included the following:

• Mild pain intensity group: 1 prolonged wound drainage without infection and 1 deep vein thrombosis (DVT).
• Moderate pain intensity group: 1 cerebrovascular event with left hemiplegia at 6 weeks post‐TKA, 1 readmission due to hypercalcemia and hyponatremia at 2 weeks post‐TKA (subsequently diagnosed with leukemia), and 1 patellar fracture after fall.
• Severe pain intensity group: highest incidence of complications (*p*  <  0.01), including 1 superficial infection, 3 DVT, 1 intracerebral hemorrhage with right hemiplegia at 10 days post‐TKA, and 1 death due to acute myocardial infarction at 3 months post‐TKA.

## 4. Discussion

Historically, TKA has been associated with significant postoperative blood loss, often necessitating allogeneic blood transfusions. Both nonmodifiable factors (e.g., advanced age and female gender) and modifiable factors (e.g., extensive surgical trauma and prolonged operative time) have been shown to increase blood loss after TKA [3, 4]. Current perioperative management strategies, including patient preconditioning, less invasive surgical techniques, multimodal pain control, TXA use, and restricted blood transfusion thresholds, have significantly reduced blood loss after TKA [5, 6, 17, 20, 21]. Consequently, the significance of previously established factors related to blood loss may have changed. Blood loss after major surgery can be affected by various determinants. Postoperative pain, which can induce hypertension or increase mean arterial pressure, has been demonstrated to influence perioperative blood loss [7, 22]. However, the correlation between postoperative pain intensity and blood loss has not been extensively explored.

Our primary outcome analysis did not demonstrate a significant effect of postoperative pain intensity on CBL. However, the severe pain intensity group tended to have a higher blood transfusion risk (risk ratio = 1.92, *p* = 0.068) compared to the mild pain intensity group. These findings contrast with a previous smaller study (*n* = 60) that reported a relationship between CBL and morphine consumption after TKA [7]. They found a positive correlation between CBL and morphine consumption at 6–12 h post‐TKA (*p* = 0.02). They also observed a significant positive correlation between measured blood loss and mean arterial pressure during physiotherapy, suggesting that inadequate pain control might increase sympathetic stimulation and mean arterial blood pressure, subsequently leading to greater blood loss. Nevertheless, a prospective study by Kim et al. [11], which included 91 patients undergoing bilateral TKA with combined spinal‐epidural anesthesia, showed no differences in postoperative mean arterial pressures at 6, 12, and 24 h among patients with mild, moderate, and severe pain intensity. They also demonstrated that blood loss at 6–24 h postoperatively did not differ among the three pain intensity groups. However, it is important to note that only visible blood loss, including intraoperative blood loss and postoperative drain volume, was analyzed, rather than total blood loss. In line with our findings, another study involving 360 patients also found no relationship between CBL and pain scores after TKA [12].

In our study, only modifiable factors, including preoperative Hb level and operative time, were positively correlated with CBL. Preoperative Hb level was the sole protective factor against blood transfusion after TKA. Multivariable regression analysis revealed no correlation between demographic characteristics (age, gender, BMI, and ASA score) and CBL or blood transfusion risk. These findings differ from previous studies reporting that age over 75 years, BMI less than 27 kg/m^2^, and higher ASA grade were associated with increased blood transfusion risk [5, 23]. Consistent with our results, Guay [7] demonstrated a positive correlation between preoperative Hb and CBL, while Pierson et al. [24] showed that preoperative Hb levels > 12 g/dL reduced the risk of blood transfusion after TKA. Furthermore, patient preconditioning with ferrous sulfate and folic acid in the 30–45 days prior to TKA led to a significantly lower rate of blood transfusion compared to the control group [20]. Similarly, a meta‐analysis found that preoperative erythropoietin (EPO) administration significantly reduced blood transfusion rates and resulted in higher Hb concentrations after lower limb arthroplasty compared to no EPO treatment [21].

Although the use of a tourniquet enhances surgical field visualization, reduces intraoperative blood loss, and shortens operating time for TKA, there was no difference between using tourniquet or not using in terms of postoperative total blood loss and transfusion rate when the TXA was applied [23]. Nonetheless, our study found that longer operative times with tourniquet application were associated with increased CBL. Moreover, prolonged use of the tourniquet has been linked to increased postoperative knee and thigh pain, thigh swelling, diminished quadriceps function, elevated inflammatory markers, and a higher incidence of local complications such as blisters [18, 25, 26]. Therefore, reducing surgical duration and avoiding prolonged tourniquet use are recommended to minimize blood loss and prevent related adverse effects.

Current studies have revealed that multimodal pain management incorporating preemptive analgesia, PNBs, periarticular injections, and intravenous NSAIDs promotes faster recovery and reduces hospital stay duration [6, 8]. Our secondary outcome analysis showed that postoperative pain intensity was not associated with LHS. However, NSAID use was an independent factor that could shorten LHS without increasing blood loss. Importantly, patients experiencing severe pain intensity after TKA had a higher rate of complications, such as DVT and cardiovascular events. This supports previous findings that inadequate pain control can increase the risk of perioperative complications such as decreased pulmonary function and lung atelectasis, venous thromboembolism, and infection [8]. In case that pain is severe, it may alter the use of oxygen in the myocardium and subsequently lead to ischemic cardiac events [10]. Currently, NSAID is widely incorporated into multimodal analgesia because evidence has shown that intravenous NSAIDs were effective in mitigating pain and opioid use, reducing opioid‐related side effects, and increasing knee joint movement after TKA [6, 8, 9]. Ketorolac, a nonselective COX inhibitor, can theoretically inhibit cyclooxygenase and affect platelet aggregation and potentially increase perioperative bleeding. Some studies have reported a 6% greater decrease in hematocrit compared to placebo at 24 h after TKA [19, 27]. However, our study did not find an effect of NSAIDs on blood loss, likely due to smaller surgical exposure and the use of TXA, which is now standard practice.

Limitations of this study include its retrospective design that may introduce potential selection bias due to uncontrolled factors in real clinical settings. Despite being performed by a single surgeon with a consistent perioperative protocol, the study cohort—limited to Asian patients and predominantly female—may reduce variability. These may conversely limit the generalizability of our findings. Second, the severe pain group is disproportionately small compared to the mild and moderate pain groups, while the moderate pain group had longer operative time than other groups. Finally, variations in the cutoff levels for blood transfusion thresholds across hospitals may affect outcomes. Thus, the clinical significance of our results may require further investigation with larger cohort of multiple institutions.

## 5. Conclusion

Patients experiencing severe pain intensity in the first 24 h after TKA tend to have a higher risk for blood transfusion and a significantly higher risk of overall complications. Additionally, incorporating NSAIDs into the treatment plan should be considered as a strategy to shorten the LHS. Thus, optimizing pain control regimen is important to enhance recovery for current clinical pathways of TKA.

## Disclosure

All the authors have read and agreed to the published version of the manuscript.

## Conflicts of Interest

The authors declare no conflicts of interest.

## Author Contributions

P.V., S.T., A.M., and A.L. contributed to the conceptualization and methodology of the study. S.P. and S.R. were responsible for data curation and investigation. All authors were responsible for data interpretation and performed the statistical analysis. P.V., S.T., and A.L. were involved in validation and project supervision and wrote the original draft of the manuscript. A.M. and A.L. reviewed and edited the manuscript.

## Funding

The authors received no specific funding for this work.

## Data Availability

Data are available on request due to privacy/ethical restrictions.
